# Upregulated VEGF and Robo4 correlate with the reduction of miR-15a in the development of diabetic retinopathy

**DOI:** 10.1007/s12020-019-01921-0

**Published:** 2019-04-12

**Authors:** Qiaoyun Gong, Fuqiang Li, Jia’nan Xie, Guanfang Su

**Affiliations:** 1 Eye Center, The Second Hospital of Jilin University, #218 Ziqiang Street, 130021 Changchun, Jilin China; 2 Department of Ophthalmology, Shanghai General Hospital, #100 Haining Road, 200080 Shanghai, China

**Keywords:** VEGF, Roundabout 4, miRNA, Diabetic retinopathy, Human retinal endothelial cell, Human retinal pigment epithelial cell

## Abstract

**Purpose:**

Vascular endothelial growth factor (VEGF) plays implicated roles in diabetic retinopathy (DR). The role of roundabout 4 (Robo 4) in angiogenesis and vasculogenesis is controversial; however, the interdependent relationship between these two factors has not been studied in DR. This study determined the colocalization of VEGF and Robo4 in fibrovascular membranes (FVM) from patients with proliferative diabetic retinopathy (PDR). MicroRNA (miRNA)-mediated modulation of VEGF and Robo4 was explored in diabetic rats and ARPE-19 tissue culture cells under hyperglycemia.

**Methods:**

VEGF and Robo4 co-expression in the FVM was analyzed using immunofluorescence. VEGF and Robo4 levels were determined in diabetic retinas and ARPE-19 tissue culture cells under high glucose using western blotting and RT-qPCR. MicroRNA agomir was intraocularly injected to increase miR-15a expression and downregulate VEGF and Robo4 levels in diabetic retinas.

**Results:**

VEGF and Robo4 colocalization in FVM vessels was observed. Increased VEGF levels were consistent in diabetic retinas and ARPE-19 tissue culture cells cultured under hyperglycemia. Robo4 decreased in ARPE-19 tissue culture cells exposed to hyperglycemia for 72 h, whereas it increased in diabetic rat retinas. Several miRNAs were differentially expressed during DR progression. Furthermore, miR-15a agomir injection inhibited high levels of VEGF and Robo4 in diabetic retinas.

**Conclusions:**

VEGF and Robo4 were co-expressed in FVMs from PDR patients. In the early stages of DR, VEGF was upregulated and contributed to DR development, whereas, in the late stage of DR, VEGF and Robo4 worked together to aggravate DR progression. However, miR-15a could downregulate VEGF and Robo4 to ameliorate DR development.

## Introduction

Diabetic retinopathy (DR) is one of the severe complications of diabetes mellitus (DM) resulting from long-term detrimental high glucose (HG) [1, 2]. DR is the leading cause of blindness in working-age adults globally and is characterized by the formation of a fibrovascular membrane (FVM) that can greatly damage patients’ visual function [3]. The blood–retinal barrier (BRB) protects and maintains retinal homeostasis by eliminating neural elements and cytotoxic reagents from circulating cells [4]. BRB is composed of inner and outer elements in which retinal microvascular endothelial cells (RECs) form the inner BRB and retinal pigment epithelial (RPE) cells form the outer BRB [5]. Exposure to hyperglycemia and the resultant damage are pivotal to the BRB imbalance and dysfunction that cause the leakage of fluids and lipids into the retina and deteriorate DR progression. Multiple pathogenic mechanisms have been explored to explain the sustained hyperglycemic effects on DR progression [1, 6, 7]. Vascular endothelial growth factor (VEGF) is involved in structural and functional changes in the retina exposed to HG or hypoxia [8, 9]. The aberrant level of VEGF aggravated the pathological angiogenesis [10] and DR development [11]. Clinical treatment has implied anti-VEGF in ocular diseases with pathological angiogenesis [12, 13]. However, not all patients with retinopathy are responsive to anti-VEGF therapy; therefore, a new effective target combined with silencing VEGF may be a more effective choice.

Roundabout 4 (Robo4) is a member of the Robo family, which is involved in angiogenesis maintenance and vessel integrity [14, 15]. Robo4 participates in physiological angiogenesis and promotes vessel maturation [16, 17]. However, in pathogenic angiogenesis, Robo4 has been found to accelerate the progression of angiogenesis [18]. In our previous study, Robo4 was found upregulated in human retinal endothelial cells under hypoxia and overexpressed in the FVMs from proliferative diabetic retinopathy (PDR) patients [19]. Recently, studies have been carried out on the interdependent roles of VEGF and Robo4 during angiogenesis. Robo4 was confirmed to negatively modulate VEGF in microvascular cells and involved in maintaining blood vessel stability and integrity in model systems [15]. Moreover, decreased Robo4 induced VEGF overexpression in diabetic cerebral neovascularization [20]. Thus the crosstalk between VEGF and Robo4 has been verified in different types of cells and diseases. However, little is known regarding the complex interaction between VEGF and Robo4 in DR.

MicroRNA (miRNA) is a group of endogenous, small non-coding RNA and modulates gene expression through transcriptional or posttranscriptional regulation [21, 22]. Several studies have detected differentially expressed miRNAs in the development of DR [23–25]. MiRNAs target hundreds of mRNA transcripts, and each mRNA can be regulated by several miRNAs. Different mRNA molecules can be modulated by the same group of common miRNAs [22]. By competing for binding to the same miRNAs, mRNA can crosstalk with another one without a direct interaction between the mRNA molecules [26]. Thus the expression of VEGF and Robo4 might be interdependent in DR via competing for their common miRNAs.

## Materials and methods

### Tissue samples

This study protocol was approved by the Ethics Committee of Jilin University, and all patients were informed according to the World Medical Association Declaration of Helsinki. A total of 24 type II DM patients with PDR were enrolled in this research of whom 11 were males and 13 females. All patients were aged 37–67 years (54.17 ± 9.28). They received pars plana vitrectomy with membrane peeling. The FVM specimens surgically obtained were fixed in 4% paraformaldehyde, then embedded in optimum cutting temperature compound (OCT), and sections cut at 3 μm.

### Animal experiments

All animal experiments were conducted in accordance with the ARVO Statement for the Use of Animals in Ophthalmic and Vision Research and approved by the Ethics Committee of the Second Hospital of Jilin University.

Male Sprague-Dawley rats (~200 g, 8 weeks old) were obtained from the Animal Center, College of Basic Medical Sciences, Jilin University. They were kept in standard plastic rodent cages and maintained in a controlled environment (24 °C, 12-h light, 12-h dark cycle). Diabetes was induced by a single intraperitoneal injection of streptozotocin (STZ; Sigma, St. Louis, MO, USA; 65 mg/kg, in citrate buffer, pH 4.5). Normal control rats received an identical volume of citrate buffer. Rats were considered diabetic when their blood glucose exceeded 16.7 mmol/L 1 week after STZ administration. Body weights of rats were also measured throughout the study. Control or diabetic rats were maintained for 0, 4, 6, and 8 weeks (*n* = 8/group) to perform further experiments. Each experiment was conducted at least three times.

### Double immunofluorescent staining

Tissue sections were rinsed in phosphate-buffered saline (PBS) three times and then permeabilized with 0.5% Triton X-100 (Beyotime, Jiangsu, China) in PBS for 15 min at room temperature. Slides were blocked with 1% bovine serum albumin in PBS for 30 min at 37 °C. Subsequently, tissues were treated with 1:150 CD34 anti-mouse polyclonal antibody (Affinity, USA) plus 1:150 VEGF anti-rabbit monoclonal antibody (Affinity, USA), 1:150 CD34 anti-rabbit polyclonal antibody (Abcam, USA) plus 1:50 Robo4 anti-mouse polyclonal antibody (Santa Cruz Biotechnology, USA), and 1:150 VEGF anti-rabbit monoclonal antibody plus 1:50 Robo4 anti-mouse polyclonal antibody, respectively, at 4 °C overnight. Meanwhile, negative control staining was conducted without primary antibody to exclude false positive fluorescence. After washing with PBS three times, the sections were then incubated with 1:600 Cy3-conjugated goat anti-rabbit (Bioss, Beijing, China) or 1:800 goat anti-mouse IgG DyLight 488-conjugated secondary antibodies (Thermo, IL, USA) for 1 h at 37 °C. Nuclei were stained with DAPI (4,6-diamidino-2-phenylindole; 1:600 diluted in PBS; Solarbio, Beijing, China). The tissue sections were observed under a fluorescent illumination microscope (Olympus IX71, Tokyo, Japan).

### Cell culture and treatment

Human retinal microvascular endothelial cells (HRECs) were purchased from ANGIO-PRO TEOMIE (Boston, MA, USA) and cultured in endothelial cell medium containing 5% fetal bovine serum (FBS) and 1% endothelial cell growth supplement (ScienCell, Carlsbad, CA, USA). The human RPE cell line ARPE-19 was obtained from American Type Culture Collection (ATCC, Manassas, VA, USA) and cultured according to the manufacturer’s instructions. The cells were maintained in Dulbecco’s modified Eagle medium/F-12 (Hyclone, Beijing, China) containing 10% FBS (Gibco; Thermo Fisher Scientific, Waltham, MA, USA). HREC and ARPE-19 cultures were maintained at 37 °C in a humidified atmosphere containing 5% CO_2_.

HRECs and ARPE-19 cells were plated at 1 × 10^4^ cells in 6-well plates (Corning; Acton, MA, USA) and treated with normal glucose (NG; 5.5 mmol/L) as a control or with HG (25 mmol/L) or the osmotic control mannitol (MN; 19.5 mmol/L MN together with NG) under normoxic conditions for 72 h to mimic the early stage of DR. The media was changed daily to eliminate metabolic byproducts and provide the nutrients necessary for the cells.

### Cell line authentication

HRECs were purchased as primary cells and the identification certificate was provided by the producer (see Supplementary Material 1). The ARPE-19 cell line was identified by STR profiling analysis, which was conducted by Shanghai Biowing Applied Biotechnology Co. Ltd. (see Supplementary Material 2).

### Western blotting

For in vivo experiments, the retinal tissues from control or diabetic rats were isolated and washed in PBS twice. For in vitro experiments, total protein was collected from HREC or ARPE-19 cells cultured under hyperglycemia. The tissues or cells were lysed for 60 min on ice in Total Protein Extraction Buffer with protease inhibitor (Transgen Biotech, Beijing, China) according to the manufacturer’s protocol and then sonicated. The lysates were collected at 12,000 rpm for 10 min at 4 °C. Protein concentrations were evaluated using a Bicinchoninic Acid Protein Assay Kit (Beyotime, Jiangsu, China). Protein lysates were electrophoresed on 10% SDS polyacrylamide gels, transferred onto polyvinylidene difluoride membranes (Millipore, USA), and then blocked in 5% skim milk for 1 h. Primary antibodies against VEGF (1:500, Affinity, USA), Robo4 (1:50, Abcam, USA) and β-actin (1:1000, CMC-TAG, USA) were applied separately at 4 °C overnight. Blots were then treated with secondary antibodies (1:5000; Boster, China) for 40 min. Finally, an Enhanced Chemiluminescence (ECL) Plus Kit (Millipore, USA) was applied for visualization. The gray bands were calculated using the Image J software.

### Total RNA isolation and reverse transcript

Total RNA was extracted from HREC and ARPE-19 cells cultured under HG condition using an Eastep Super Total RNA Extraction Kit (Promega, Shanghai, China) following the manufacturer’s protocol. The concentration and purity of RNA were measured using a NanoDrop 2000c Spectrophotometer (Thermo Fisher Scientific, Waltham, MA, USA). An A260/A280 value of approximate 2.0 was generally accepted for further analysis. The integrity of RNA samples was assessed using 1% agarose gel electrophoresis.

For analysis of VEGF and Robo4, 800 ng total RNA was reverse transcribed into cDNA using a Perfect Real Time RT Reagent Kit (Takara Bio, Dalian, China) in a 20 μL reaction volume. To analyze miRNAs levels, 1000 ng total RNA was polyadenylated and reverse transcribed by TransScript Green miRNA RT SuperMix (Transgen Biotech, Beijing, China).

### Real-time quantitative PCR (RT-qPCR)

The cDNA reverse transcribed from different samples was processed for qPCR analysis. To quantify the levels of VEGF and Robo4 mRNAs, 10 μmol gene-specific primers (forward and reverse mixed together), and 2× Fast SYBR Green Master Mix (Roche Diagnostics, Switzerland) were applied for the PCR reaction. The data were normalized to the expression of housekeeping gene glyceraldehyde 3-phosphate dehydrogenase. Primer sequences are listed in Table 1. The cDNA for analyzing miRNAs was diluted 1:5 for further qPCR with specific primers using TransScript Green miRNA qPCR SuperMix (Transgen Biotech, Beijing, China). Three replicates were performed for each biological replicate. The mature miRNAs sequences are shown in Table 2. The qPCR primers against mature miRNAs were purchased from GeneCopoeia (Rockville, MD, USA), and the expression levels of miRNAs were normalized to that of U6 expression. In each primer, two negative controls were included with ddH_2_O instead of the template. The relative expression levels of mRNAs or miRNAs were calculated using the 2^−ΔΔCt^ method, which was based on the ratio of gene expression between an experimental and control group.

**Table 1:** Primer sequences of genes

Gene subtype	Oligonucleotide primers (5′–3′)	Product size (bp)
Human VEGFA	F: GCACCCATGGCAGAAGGAGGAG R: GTGCTGACGCTAACTGACC	156
Human Robo4	F: CCCTGTGCTTGGAACTCAGTG R: CGCTGATGTACCCATAGGTGG	102
GAPDH	F: TGCACCACCAACTGCTTAGC R: GGCATGGACTGTGGTCATGAG	70

*GAPDH* glyceraldehyde 3-phosphate dehydrogenase, *Robo4* roundabout 4, *VEGFA* vascular endothelial growth factor A

**Table 2:** Mature sequence of the detected miRNAs

miRNA	Mature sequence (5′–3′)
hsa-miR-15a-5p	UAGCAGCACAUAAUGGUUUGUG
hsa-miR-16-5p	UAGCAGCACGUAAAUAUUGGCG
hsa-miR-195-5p	UAGCAGCACAGAAAUAUUGGC
hsa-miR-424-5p	CAGCAGCAAUUCAUGUUUUGAA
hsa-miR-497-5p	CAGCAGCACACUGUGGUUUGU

*miRNA* microRNA

### Intraocular injection of miRNA agomir

Agomir is a small double-stranded RNA that is labeled and chemically modified to regulate the biological function of target genes by mimicking endogenous miRNA. Agomir is especially suitable for animal experiments as it has higher stability and inhibition effects in in vivo experiments. After the onset of STZ-induced diabetes for 4 weeks, miRNA agomir (GenePharma, Shanghai, China) for miR-15a and negative control (scrambled agomir) were intravitreally injected with the Lipofectamine Reagent (Invitrogen, Carlsbad, CA, USA). Normal control rats were injected with the same volume of PBS. The animals were euthanized after treatment for 1 week, and the retinal tissues were collected. The miR-15a agomir was injected intravitreally at 200 nmol/200 µL, and the control diabetic rats received the same dose of scrambled agomir. The mature miR-15a agomir sequence is: 5′- UAGCAGCACAUAAUGGUUUGUG-3′, the scrambled agomir sequence is: 5′- UUCUCCGAACGUGUCACGUTT-3′.

### Statistical analysis

All experiments were conducted at least three times. Results were presented as the mean ± SD of three independent experiments. Student’s *t* test or analysis of variance followed by Student–Newman–Keuls post hoc test were performed using the Prism 6.0 software (GraphPad Software, San Diego, CA, USA) to assess statistical differences. Values of *p* < 0.05 are considered statistically significant.

## Results

### VEGF and Robo4 co-expression in FVM microvessels

**Fig. 1:**
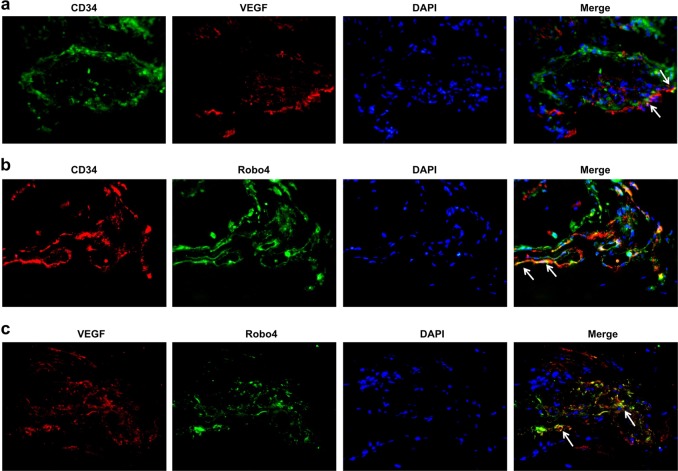
Colocalization of vascular endothelial growth factor (VEGF) and roundabout 4 (Robo4) in the fibrovascular membranes (FVMs) from proliferative diabetic retinopathy patients. **a** Double immunofluorescent staining of VEGF (red) and CD34 (green) showed colocalization of VEGF and CD34 in the blood vessels of FVMs (indicated by a white arrow). **b** Staining of Robo4 and CD34 by anti-Robo4 (green) and CD34 (red) antibody, respectively. The immunofluorescence showed Robo4 expressed in the vessels of FVMs (indicated by a white arrow). **c** Double staining of VEGF and Robo4 confirmed the colocalization in the FVMs (indicated by white arrow). **a**–**c** Original magnification: ×40

To investigate the interdependent role of VEGF and Robo4 in DR, we first determined whether VEGF and Robo4 were colocalized in FVMs by double immunofluorescence staining. Consistent with a previous study [27], VEGF was detected and expressed in the vessels of FVMs (Fig. 1a), marked with anti-CD34 antibody (recognized as a microvascular endothelial cell marker [28, 29]). Meanwhile, Robo4 was also found located in the vessels of FVMs, co-expressed with CD34 (Fig. 1b). To exclude a false positive signal, we conducted separate negative experiments without primary antibody incubation. There was no fluorescence detected in negative controls, and the nucleus was stained with DAPI (Supplementary Material Fig. 1). Further confirmation was performed where VEGF and Robo4 were colocalized and distributed in the FVMs around the vessels (Fig. 1c). These results revealed that VEGF and Robo4 co-expressed in the vessels of FVMs, and Robo4 may play a role in the formation of FVM in diabetic patients.

### Enhanced levels of in vivo VEGF and Robo4 in diabetic retinas

**Fig. 2:**
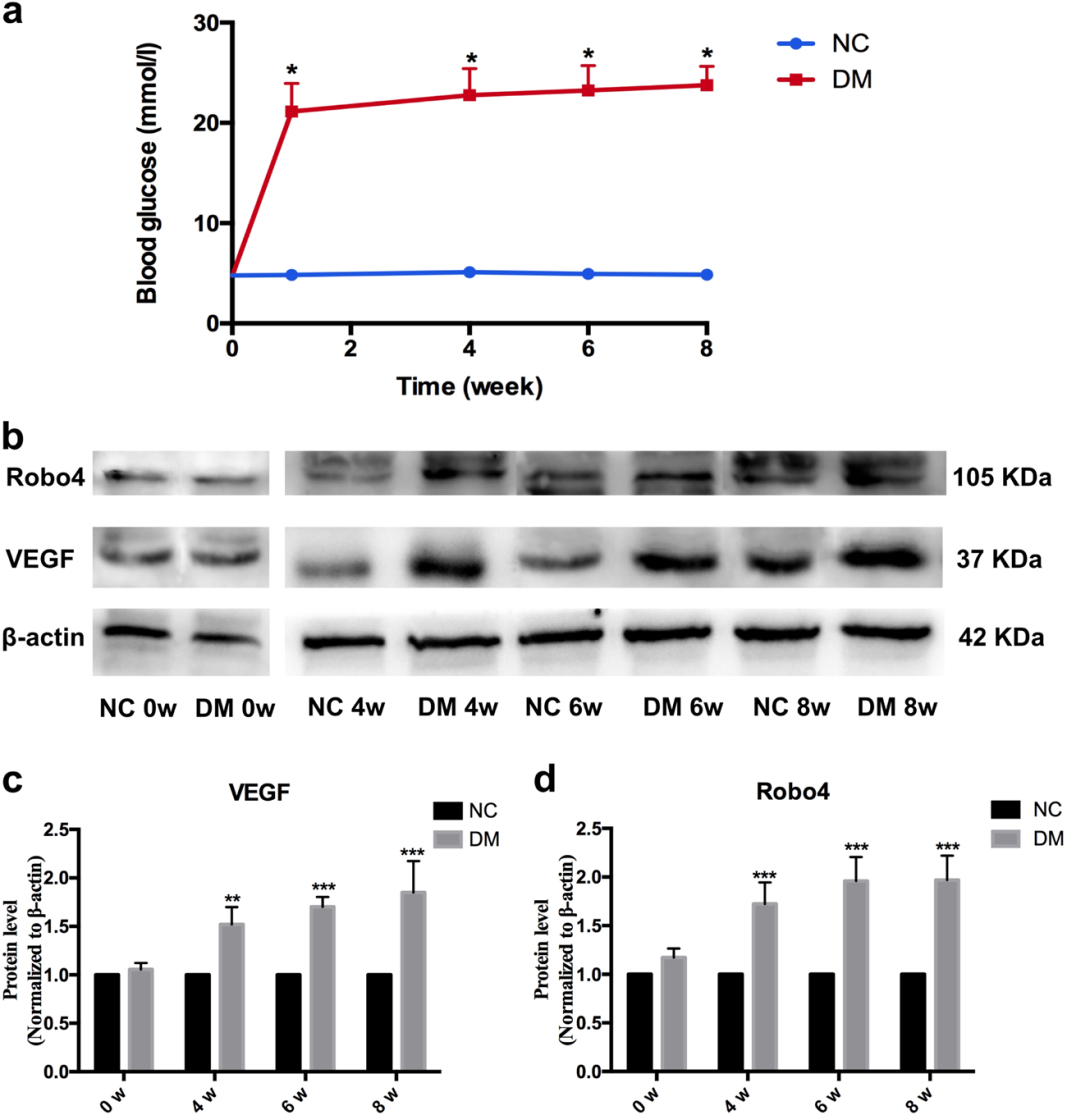
Protein levels of vascular endothelial growth factor (VEGF) and roundabout 4 (Robo4) in the retinas of diabetic rats. **a** Blood glucose levels of normal control (NC) and diabetic rats DM. (**b**) Western blots of VEGF and ROBO4 expression in the diabetic retinas after 0, 4, 6, and 8 weeks. β-Actin was used as a loading control. **c** The quantitative level of VEGF was increased in a time-dependent level with the diabetes deterioration. **d** The quantification of Robo4 confirmed the enhanced level induced by diabetes in the retinas. Bars, means ± SDs. *p < 0.05, **p < 0.01, ***p < 0.001 versus the respective negative control group (*n* = 6)

Based on the results from FVM detection, a STZ-induced diabetic animal model was used to confirm the levels and correlation of VEGF and Robo4 in the retinas. The DM rats exhibited significant weight loss compared with age-matched control rats. Furthermore, blood glucose levels were markedly elevated in STZ-treated rats after the onset of DM and sustained at a high level across the DR progression from week 4 to 8 (Fig. 2a).

Retinal tissues from diabetic or control rats were used for protein validation through western blotting analysis. The protein level of VEGF elevated after DM onset for 4 weeks and then continued increasing from week 6 to week 8 (Fig. 2b, c). It can be implied that the expression of VEGF was about two-fold higher than control rats. Similarly, Robo4 was overexpressed in diabetic retinas after 4 weeks of uncontrolled diabetes, and the high level was kept to week 8 (Fig. 2b, d). It was significant that Robo4 was about two-fold higher than that in normal retinas. These results confirmed that VEGF and Robo4 were enhanced in DR progression, and in return, their high levels deteriorated DR development.

### HG induced VEGF overexpression by downregulating Robo4 in vitro

**Fig. 3:**
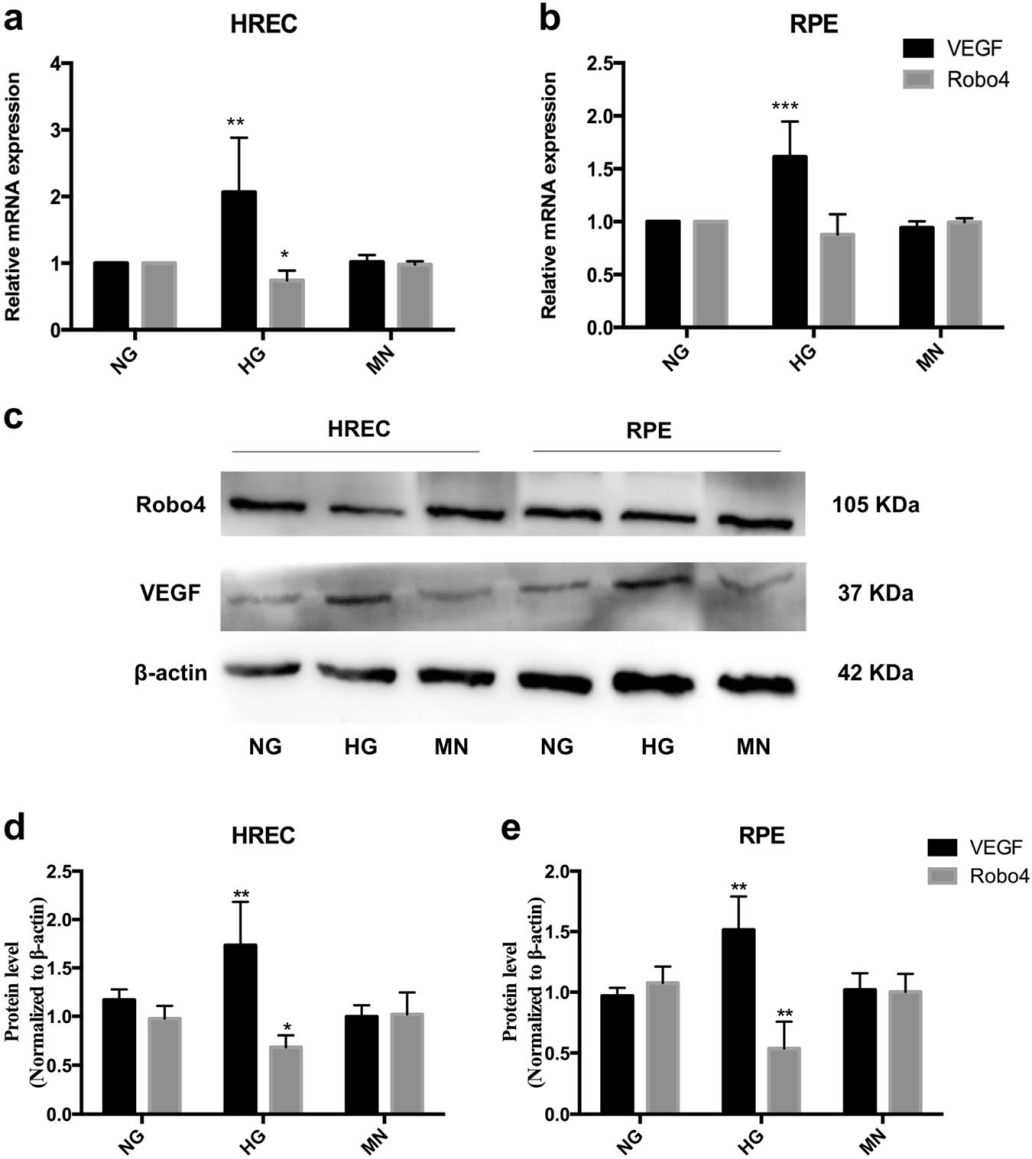
Vascular endothelial growth factor (VEGF) and roundabout 4 (Robo4) expression in human retinal microvascular endothelial cells (HRECs) and retinal pigment epithelium (RPE) cells under hyperglycemia. **a**, **b** The mRNA levels of VEGF and Robo4 in HRECs and RPE cells exposed to high glucose. VEGF was upregulated significantly, whereas Robo4 was inhibited. No change was observed in mannitol control groups. **c**–**e** Western blots analysis showed the protein levels of VEGF and Robo4 in HRECs and RPE cells under hyperglycemic conditions. Similarly with transcript variation, VEGF was induced and Robo4 was decreased by high glucose. β-Actin was used as a loading control. Bars, means ± SDs. *p < 0.05; **p < 0.01; ***p < 0.001 versus the respective negative control group (*n* = 6)

To investigate the molecular changes induced by HG and assess the effects of glucose on the retina, we conducted in vitro studies using HRECs and RPE cells cultured under hyperglycemic conditions. The expression of VEGF and Robo4 in HRECs and RPE cells was confirmed by RT-qPCR and western blotting. On the mRNA level, HG induced overexpression of VEGF and inhibited the expression of Robo4 after 72 h in HRECs and RPE cells (Fig. 3a, b). Osmotic mannitol control did not cause any change in VEGF and Robo4 expression. Consistent with mRNA changes, the protein levels of VEGF were elevated in HREC and RPE cells exposed to hyperglycemia, with the downregulation of Robo4 (Fig. 3c–e). Increases in VEGF of >1.5-fold was observed, and decreases in Robo4 of 0.5-fold were found in the hyperglycemic group. Interestingly, in the early and short-term exposure to HG, increased VEGF was accompanied by Robo4 downregulation in retinal cells. Thus the interaction between VEGF and Robo4 may be differed in the progression of DR via different modulation.

### The aberrantly expressed miRNAs targeting VEGF in HREC and RPE cells exposed to hyperglycemia

**Fig. 4:**
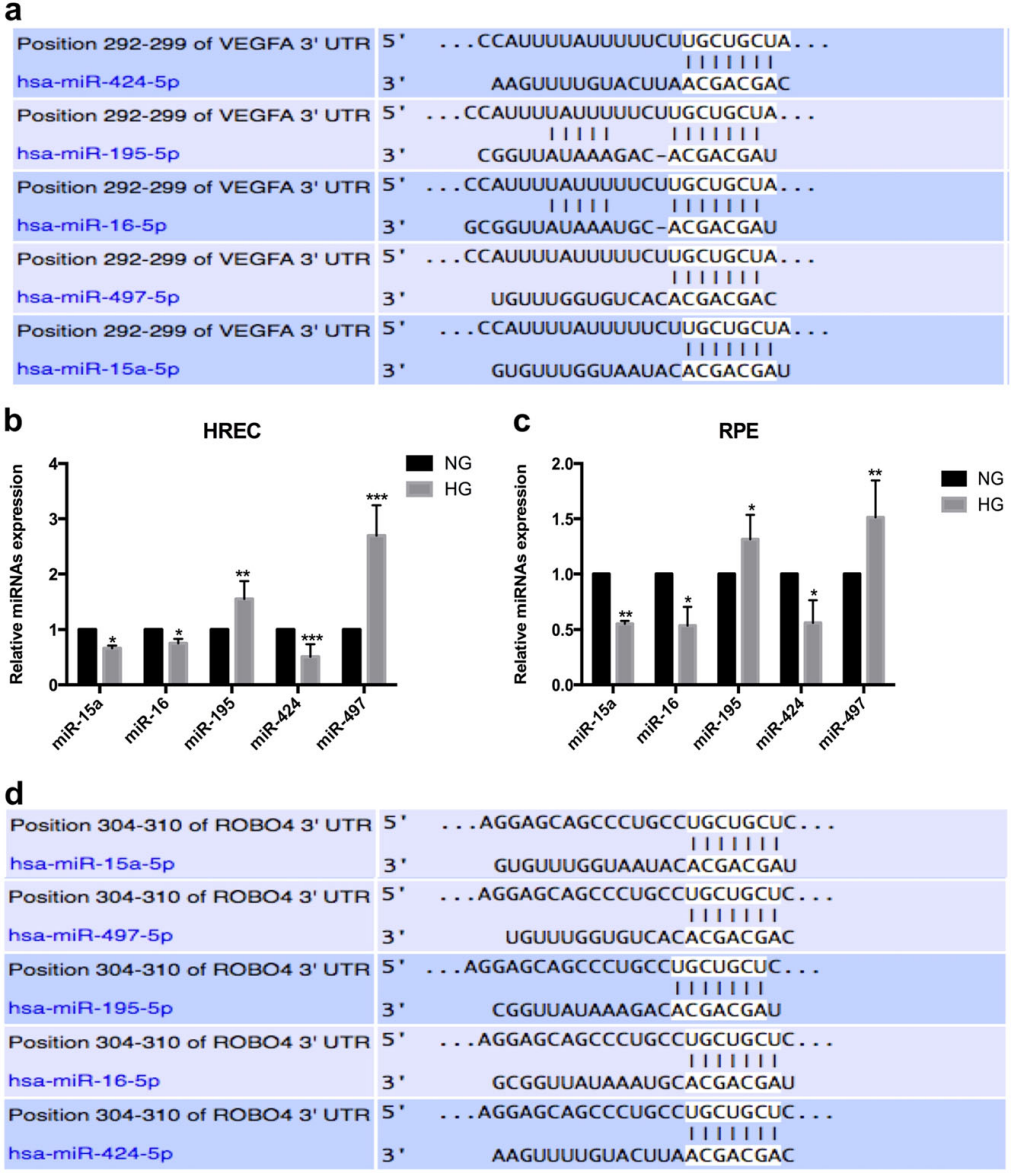
Differentially expressed microRNAs (miRNAs) targeting vascular endothelial growth factor (VEGF) and roundabout 4 (Robo4) in human retinal microvascular endothelial cells (HRECs) and retinal pigment epithelium (RPE) cells under hyperglycemia. **a** Potential binding sites of miRNAs on the 3’-untranslated region (3’-UTR) of VEGF predicted by TargetScan. **b** miRNA expression in HRECs under high glucose. **c** The variations of miRNAs in RPE cells exposed to hyperglycemia. **d** The potential binding sites of miRNAs on the 3’-UTR of Robo4 predicted by TargetScan. *p < 0.05; **p < 0.01; ***p < 0.001 versus the respective negative control group (*n* = 4)

We used TargetScan 7.2 (www.targetscan.org) and found that the miR-15 family (including five potential miRNAs) may affect VEGF (Fig. 4a). The expression levels of these miRNAs were determined by qRT-PCR in HREC and ARPE-19 cells exposed to HG in vitro. The results exhibited that the expression of miR-15a, miR-16, and miR-424 in HREC and RPE under hyperglycemia was decreased and the expression of miR-195 and miR-497 was increased (Fig. 4b, c). Further bioinformatics analysis showed that miR-15a family may also target Robo4 directly (Fig. 4d). Combined with previous results, miR-15a was chosen and implied as a potential therapeutic target to inhibit the expression of VEGF and Robo4 in the following study.

### miR-15a-5p inhibits VEGF and Robo4 in diabetic rats in vivo

**Fig. 5:**
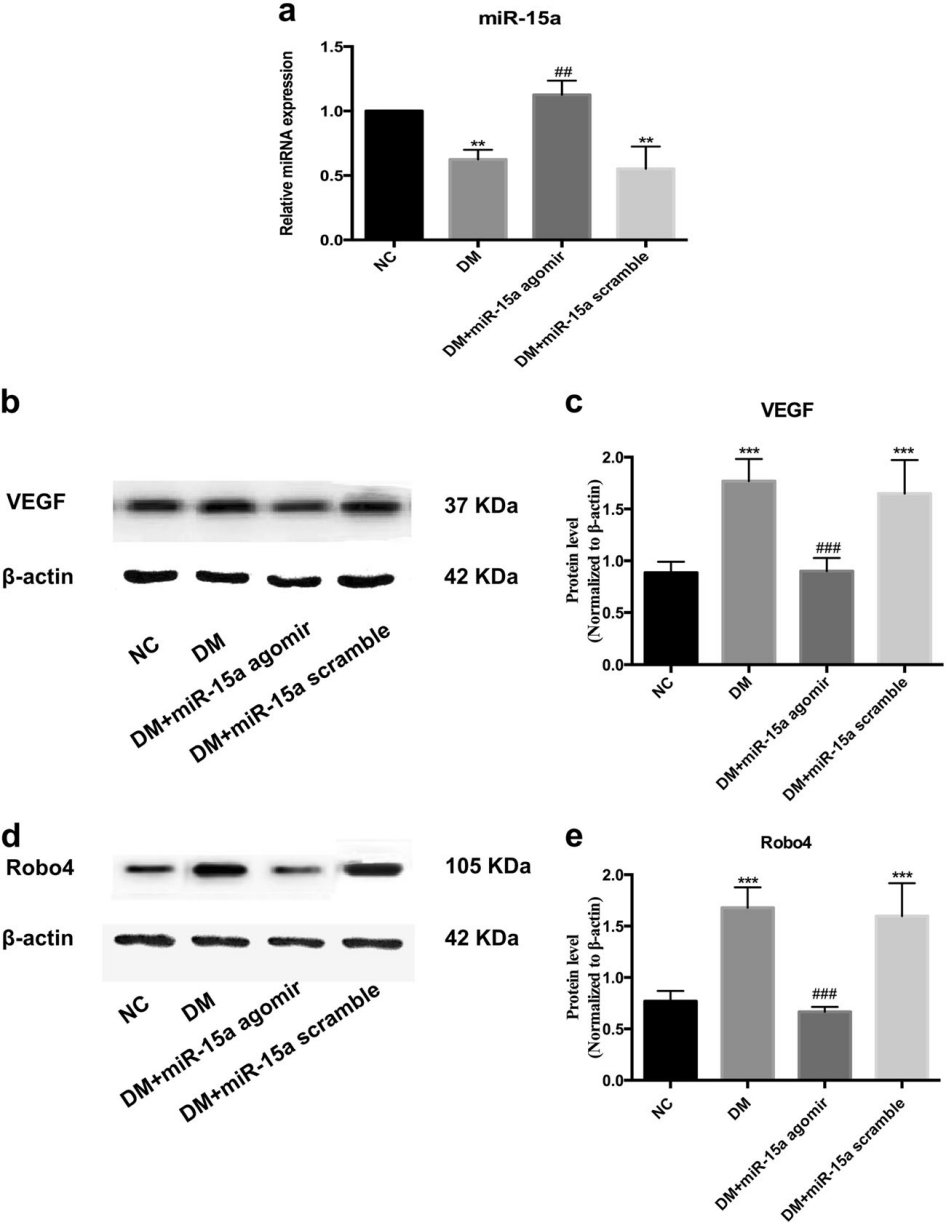
Regulatory effect of miR-15a on vascular endothelial growth factor (VEGF) and roundabout 4 (Robo4) in vivo. **a** The downregulated expression of miR-15a in diabetes mellitus retinas was rescued by miR-15a agomir injection. **b**, **c** The enhanced protein level of VEGF was inhibited by miR-15a upregulation in diabetic retinas. **d**, **e** The elevated expression of Robo4 induced by diabetes was decreased to normal levels via miR-15a agomir injection. Bars, means ± SDs. **p < 0.01; ***p < 0.001 versus the respective negative control group. ^##^*p* < 0.01; ^###^*p* < 0.001 versus the diabetes group (*n* = 6). Each experiment was conducted at least four times

To test whether miR-15a-5p can inhibit both VEGF and Robo4 protein levels, we detected the regulation in an in vivo animal model of DM. Diabetes obviously inhibited the level of miR-15a in the rat retinas (Fig. 5a). After onset of diabetes for 1 month, miRNA agomir was injected intraocularly and sustained for a week. The injection of miRNA agomir significantly enhanced miR-15a expression to normal levels, whereas the scramble agomir control did not cause any change where miR-15a was downregulated in the DM retinas (Fig. 5a). Overexpressing miR-15a prohibited the protein levels of both VEGF and Robo4 compared with that of the scramble control in DM rats (Fig. 5b–e). The decreased levels of VEGF and Robo4 were close to those in the normal rat retinas. The results suggested that miR-15a could effectively inhibit VEGF and Robo4 expression, and thus miR-15a may be a potential new therapeutic target in DR treatment.

## Discussion

DR is an ophthalmic microvascular complication of diabetes and results from long-term detrimental hyperglycemia [2, 3]. Hyperglycemia induces inflammation, oxidative stress, apoptosis, and accumulation of glycosylated end products [30, 31]. Constantly stimulated by these changes, the pre-exiting vessels in the retina abnormally proliferate and migrate to form new blood vessels, contributing to retinal neovascularization (RNV). This pathogenic neovascularization is deficient in tight junctions and causes plasma to leak into surrounding tissue, including vitreous. As a result, vitreous hemorrhage is induced and it attributes to the FVM formation in PDR. One or more angiogenic factors released by retina under HG condition contribute to RNV. It has been recognized that VEGF is related to structural and functional changes in the retina under diabetic conditions [8, 9]. Thus VEGF attributes to pathogenic angiogenesis [10] and DR [11]. The use of anti-VEGF treatments in DR is a pre-operation choice. A recent study showed that Robo4, an endothelial-specific marker, is involved in maintaining the blood vessel stability and integrity by counteracting VEGF and inhibiting angiogenesis in the model system [15]. Moreover, it has been reported that Robo4 interacts with UNC5B and sustains the vascular integrity by inhibiting VEGF signaling in endothelial cells [32]. Lately, Abdelsaid et al. found that, in cerebral neovascularization of diabetes, VEGF was enhanced and accelerated the progression by the downregulation of the guidance protein Robo4 [20]. Therefore, the interdependent effects between VEGF and Robo4 are diverse in different kinds of cells and diseases. However, there has been no study, to the best of our knowledge, on how VEGF and Robo4 interact in DR.

In the present study, we analyzed the FVMs to identify the positive expression and colocalization of VEGF and Robo4 using immunofluorescence. CD34, a vascular endothelial marker, confirmed that VEGF and Robo4 were expressed around the vessels of FVMs, consistent with the results of a previous study [33]. This suggested that VEGF and Robo4 were involved in angiogenesis and contributed to the formation of FVM in PDR progress. To further explore the interaction between VEGF and Robo4 in DR development, we detected the gene changes in vivo in the diabetic retinas and in vitro in hyperglycemic HREC and RPE cells. As the duration of uncontrolled diabetes was prolonged, VEGF and Robo4 expression were enhanced and sustained at a high level in the retinas during the DR progression. Furthermore, VEGF was increased in HREC or RPE cells exposed to HG. Nevertheless, Robo4 expression decreased in HREC or RPE under hyperglycemia. According to the above results and previous studies, we assumed that the interaction of VEGF and Robo4 in DR has three stages: (1) in a normal physiological state, Robo4 inhibits VEGF expression to stabilize the vascular network; (2) under the early pathogenic stage, such as HG, VEGF expression may be increased by inhibiting Robo4, resulting in DR; (3) in the course of DR, VEGF and Robo4 expression was increased, and they worked together to promote DR progression. Thus, in late DR, VEGF and Robo4 were enhanced and could attribute to deteriorating DR. Determining a target that can modulate both VEGF and Robo4 may be an effective therapy in DR treatment.

miRNAs are small, non-coding RNAs that can bind to the 3’-untranslated region of target mRNAs to induce mRNA degradation or inhibiting protein translation [21, 22]. An increasing number of miRNAs has been identified to aberrantly express and play important roles in DR progression [23, 24, 34–36]. To compete for common miRNAs, protein-coding mRNAs may crosstalk with others without direct binding [26]. Here we explored several miRNAs targeting VEGF first in HREC and RPE cells under hyperglycemia. Initial results obtained from in vitro studies demonstrated that miRNAs were markedly dysregulated under HG. Among the identified miRNAs, miR-15a, miR-16, and miR-424 were decreased, whereas miR-195 and miR-497 were increased. Considering that VEGF was enhanced and Robo4 was inhibited in HREC and RPE cells under hyperglycemia, we focused on analyzing miR-15a, miR-16, and miR-424. Further bioinformatics predicted that miR-15a may also target Robo4. This prediction verified that, when HREC and RPE cells were exposed to hyperglycemia, enhanced VEGF inhibited Robo4 via the downregulation of miR-15a. Then the regulatory role of miR-15a in VEGF and Robo4 during DR was detected in vivo in DM rats. miR-15a overexpression significantly inhibited the high levels of VEGF and Robo4 in the diabetic retinas and were then reduced to normal expression. Therefore, miR-15a may be applied to ameliorate DR progression by downregulating VEGF and Robo4. Reviewing previous studies, miR-15a has regulatory effects on insulin resistance [37] and also plays inhibitory roles in proinflammatory signaling, reducing retinal leukostasis in DR [38]. Moreover, Wang et al. reported on the dual anti-inflammatory and anti-angiogenic actions of miR-15a in DR [39]. In this study, in the animal model of overexpressed miR-15a, acid sphingomyelinase and VEGFA levels were directly reduced to nondiabetic levels; diabetes-induced increased retinal permeability was also prevented in these mice. These studies confirmed that miR-15a can modulate multiple pathologies in DR. Thereby, miR-15a can be a therapeutic target to treat DR through several pathways, including inhibiting VEGF and Robo4. Generally, in the late stage of DR, VEGF and Robo4 had synergistic effects on FVM formation and promoted the progression of the disease. In the present study, we provided the first evidence that VEGF and Robo4 are potential targets of miR-15a, and miR-15a overexpression significantly inhibits VEGF and Robo4 in DR progression.

## Supplementary information

Supplementary Material 1-HREC

Supplementary Material 2-ARPE

## Abbreviations

DR: diabetic retinopathy
DM: diabetes mellitus
FVM: fibrovascular membrane
BRB: blood–retinal barrier
REC: retinal microvascular endothelial cell
RPE: retinal pigment epithelium
VEGF: vascular endothelial growth factor
Robo4: roundabout 4
miRNA: MicroRNA
STZ: streptozotocin
HREC: human retinal microvascular endothelial cell
NG: normal glucose
HG: high glucose
MN: mannitol
RNV: retinal neovascularization
3′-UTR: 3′-untranslated region
PDR: proliferative diabetic retinopathy
