# Supplementary material for: Upregulated VEGF and Robo4 correlate with the reduction of miR-15a in the development of diabetic retinopathy
Source: Endocrine. 2019 Apr 12;65(1):35–45. doi: 10.1007/s12020-019-01921-0 (PMC6606763; doi:10.1007/s12020-019-01921-0)
Supplement: Supplementary file 1 — Supplementary Material 1-HREC [file 12020_2019_1921_MOESM1_ESM.pdf]

DATA SHEET

Human Retinal Microvascular Endothelial Cells

ORDER INFORMATION

Name of Cells: Human Retinal Microvascular Endothelial Cells **(HRMVECs)**
Catalogue Number: **cAP-0010**
Product Format: Proliferating cells in T25 or a Frozen Vial
Cell Number: > 90% confluent in T25 flask or Frozen Vial (>5x 10^5/vial)

General Information
HRMVECs (**cAP-0010**) are initiated by elutriation from dissociated normal human retinal tissue. The cells are shipped in proliferating culture with >90 confluence (the cells are provided @ passage 3). Endothelial Growth Medium (EGM, contains 5% serum and growth supplements, cAP-02) is recommended for cell culture and these cells have a minimum average population doubling levels > **20** when cultured following the detailed protocol described below).

Characterization of the cells

Cytoplasmic VWF / Factor VIII:	**>95% positive by immunofluorescence**
Cytoplasmic uptake of Di-I-Ac-LDL:	**>95% positive by immunofluorescence**
Cytoplasmic PECAM1	**>95% positive by immunofluorescence**

HRMECs are negative for HIV-1, HBV, HCV, and mycoplasma.

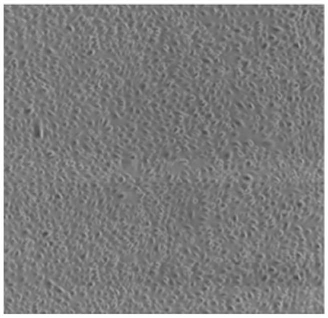

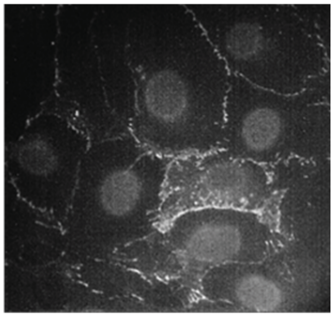

Phase contrast of immunocytochemistry stain of PECAM1 of HRMECs

Product Use: HRMVECs are for research use only.

Shipping: Proliferating culture in T25 flask or Proliferating cells in T25 or a Frozen Vial.

Handling of Arriving Cells

(1) When you receive the cells in a T25 flask, leave the T25 flask in 37°C CO2 incubator for 1 hour first, and then replace the transport medium with fresh EGM full medium. Let the cells grow for 24 hour before subculture.

(2) When you receive the cells in a frozen vial, you can transfer the vial of cells into a -80ºC freezer for short period storage or a liquid nitrogen tank for long term storage. Thaw the cells in a 37°C water bath,

11 Park Drive, Boston, MA 02215, USA | Tel: 617-549-2665 | Fax: 480- 247-4337 | E-mail: angioproteomie@gmail.com

and then transfer the cells into a T25 flask pre-coated with Quick coating solution (cAP-01) as described in details in Subculture Protocol.

Subculture Protocol

A) Pre-coating of T25 flasks: Add 2ml of Quick Coating Solution (**cAP-01**) into one T25 flask and make sure whole surface of the flask is covered with the coating solution. Five minutes later, dispose excessive Quick Coating Solution by aspiration and the flask is ready to be used (no need for overnight incubation when using Quick Coating Solution). Other extracellular matrix can be used including gelatin, collagen, and fibronectin and you are advised to test the conditions for using those materials in advance.

B) Rinse the cells in T25 flask with 5ml HBSS (**Room Temperature, RT**) twice.

C) Add 2ml of Trypsin/EDTA (**RT**) (cAP-23) into one T25 flask (make sure the whole surface of the T25 flask is covered with Trypsin/EDTA), and gently dispose the excessive Trypsin/EDTA solution **within 20 seconds** with aspiration.

D) Leave the T25 flask with the cells at **RT** for 1 minute (the cells usually will detach from the surface within 1-2 minutes). You can monitor the cells under microscope and when most of cells become rounded up, hit the flask against the bench surface, and the cells will move on the surface of the flask when monitoring under microscope.

E) Add 5ml Trypsin Neutralization Buffer and spin the cells down with 800g for 5 minutes.

F) Re-suspend the cell pellet with 10 - 20ml of EGM full medium and the cell suspension is transferred directly into 2 or 4 pre-coated T25 flasks (5ml each, and the cells are sub-cultured at 1:2 to 1: 4 ratios)

G) Change medium every 2-3 days and cells usually become confluent within 7 days (when split at a 1:4 ratio).

H) If you need prepare quiescent cells, when cells are almost confluent, replace EGM full medium with Endothelial Basal Medium (EBM, cAP-03) containing 0.5% FBS about 8-12 hours before your experiments.

Related products

Quick Coating Solution	cAP-01	240ml	Angio-Proteomie
Endothelial Growth Medium	cAP-02	500ml	Angio-Proteomie
Endothelial Basal Medium	cAP-03	500ml	Angio-Proteomie
HBSS w/o Ca^{2+} , Mg^{2+}	cAP-11	100ml	Angio-Proteomie
Trypsin/EDTA Solution	cAP-23	100ml	Angio-Proteomie
Trypsin Neutralization Solution	cAP-28	100ml	Angio-Proteomie

Caution: Handling human tissue derived products is potentially bio-hazardous. Although each cell strain is tested negative for HIV, HBV and HCV DNA, diagnostic tests are not necessarily 100% accurate; therefore, proper precautions must be taken to avoid inadvertent exposure. Always wear gloves and safety glasses when working these materials. Never mouth pipette. We recommend following the universal procedures for handling products of human origin as the minimum precaution against contamination.

11 Park Drive, Boston, MA 02215, USA | Tel: 617-549-2665 | Fax: 480- 247-4337 | E-mail: angioproteomie@gmail.com
