# Supplementary material for: Upregulated VEGF and Robo4 correlate with the reduction of miR-15a in the development of diabetic retinopathy
Source: Endocrine. 2019 Apr 12;65(1):35–45. doi: 10.1007/s12020-019-01921-0 (PMC6606763; doi:10.1007/s12020-019-01921-0)

SHANGHAI BIOWING APPLIED BIOTECHNOLOGY Co. LTD

Cell Line Authentication – STR Profiling

Sample Type: Cell Line

Sample from: Jilin University

Testing Method: STR Genotyping

Report Time: October 24, 2016

COMPANY STATEMENT

1. THIS REPORT IS ONLY RESPONSIBLE FOR THE SAMPLES ANALYZED.
2. THE TESTING RESULTS AND THE ORGANIZATION NAME WILL NOT BE USED FOR ADVERTISEMENT, COMMERCIAL EXHIBITIONS, COMMERCIAL PERFORMANCE AND OTHER COMMERCIAL ACTIVITIES.
3. OBJECTIONS SHOULD BE RAISED WITHIN FIFTEEN DAYS AFTER THE RECEIPT OF THIS REPORT.
4. THE PAPER REPORT WITH CONTENT ALTERING, ADDING OR WITHOUT THE STAMPED SEAL OF THE COMPANY ARE INVALID.

Testing Company: Shanghai Biowing Applied Biotechnology Co. Ltd

Address: Room 4F, 8 th Buiding,Guiguo Garden,NO.471 Guiping Road,Caohejing Development Zone,Shanghai

Tel: +86-021-33559491

Contact: YiQun Chen

E-mail: biowing@vip.163.com

Cell Line Authentication – STR Profiling Report

Sample code

Table 1. Sample Code

Customer's code	Company Code
ARPE-19	20161019-01

Sample Number:1

Sample Type: Cell line

Testing Type: STR

Sample From: Jilin University

Testing Method:

DNA was extracted by a commercial kit from Axygen (AP-MN-BL-GDNA-250G). The twenty STRs including Amelogenin locus were amplified by six multiplex PCR and separated on ABI 3730XL Genetic Analyzer. The signals were then analyzed by the software GeneMapper.

Data Interpretation:

Cell lines were authenticated using Short Tandem Repeat (STR) analysis as described in 2012 in ANSI Standard (ASN-0002) by the ATCC Standards Development Organization (SDO) and in Capes-Davis et al., Match criteria for human cell line authentication: Where do we draw the line? Int J Cancer.2013;132(11):2510-9.

Test Results:

1. Result

Table 2. Matching information on the cell lines

Sample Code	Multi-allele	Cell line matched	Cell Bank	Percentage
20161019-01	no	ARPE-19	DSMZ	9/9

- **Multi-allele means some STR contain more than two loci.**

2. Sample Description

20161019-01: The DNA of the cell lines found to perfect match the type of cell lines in a cell line retrieval, DSMZ database shows that cells called **ARPE-19**, corresponding to the cell number **CRL-2302.** No multiple alleles were found in this cell line.

Note: We use DSMZ tools to carry on the cell line comparison, which contains 2455 cell lines STR data from ATCC, DSMZ, JCRB, ECACC and RIKEN databases. If the cell is not included in the above cell library, users need to compared with other databases.

3. Genotyping Result

Table 3. STR and Amelogenin Genotyping Results of Cell line 20161019-01

Marker	Sample				Cell Bank information		
	Allele1	Allele2	Allele3	Allele4	Allele1	Allele2	Allele3
D5S818	13	13			13	13	
D13S317	11	12			11	12	
D7S820	9	11			9	11	
D16S539	9	11			9	11	
VWA	16	19			16	19	
TH01	6	9.3			6	9.3	
AMEL	X	Y			X	Y	
TPOX	9	11			9	11	
CSF1PO	11	11			11	11	
D12S391	21	22					
FGA	23	23					
D2S1338	19	19					
D21S11	28	29					
D18S51	12	16					
D8S1179	13	13					
D3S1358	14	15					
D6S1043	17	19					
PENTAE	7	11					
D19S433	12	13					
PENTAD	11	13					

The allele match algorithm compares the 8 core loci plus amelogenin only, even though alleles from all loci will be reported when available.

Others:

1. Genotyping Strategy and Site Distribution

Attached Table. Experimental Strategy and Sites

	**Strategy 1**	**Strategy 2**	**Strategy 3**	**Strategy 4**	**Strategy 5**	**Strategy 6**
1	TH01	AMEL	TPOX	D3S1358	PENTAE	D19S433
2	D12S391	D5S818	VWA	D13S317		PENTAD
3	D7S820	D2S1338	D8S1179	D6S1043		
4	CSF1PO	D21S11		D16S539		
5	FGA	D18S51				

2. DSMZ tools was used to carry on the cell line comparison, which contains 2455 cell lines STR data from ATCC, DSMZ, JCRB ,ECACC and RIKEN databases. If the cell is not included in the above cell library, users need to compared with other databases.

3. Reference

[1]Authentication testing of HEK 293T and HeLa cell lines have been performed by Shanghai Bio wing Applied Biotechnology Co.,Ltd via STR profiling. STR profiles match the standards recomm ended for HEK 293T and HeLa cell lines authentication

[2]AGS, NCI-N87, HGC-27 and HEK293 were STR-authenticated on Dec. 8, 2015 by Shanghai Biowing Applied Biotechnology Co. LTD, Shanghai, China

Technician: Menglu Shen , Jianan Zhang
Check: Yang Bai
Person in Charge: Yiqun Chen

Issue date: October 24, 2016

Applied Biosystems
GeneMapper 4.0

Cell Line Authenticaion-4

Sample File	Sample Name	Panel	OS	SQ
47_G06_CellLineAuthentication-1-1019.fsa	ARPE-19	STR Profile 1-dup		

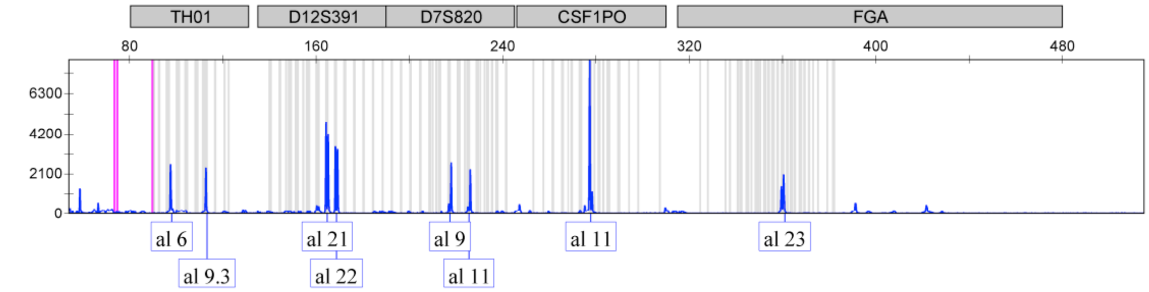

Sample File	Sample Name	Panel	OS	SQ
51_C07_CellLineAuthentication-5-1019.fsa	ARPE-19	STR Profile 2-dup		

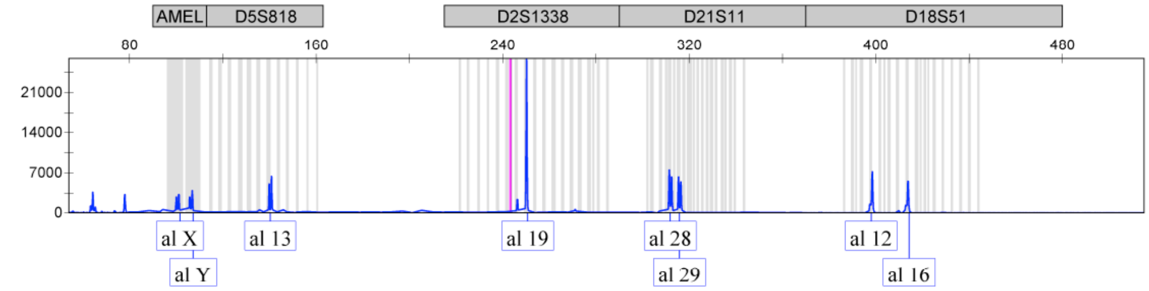

Sample File	Sample Name	Panel	OS	SQ
58_B08_CellLineAuthentication-5-1019.fsa	ARPE-19	STR Profile 3-dup		

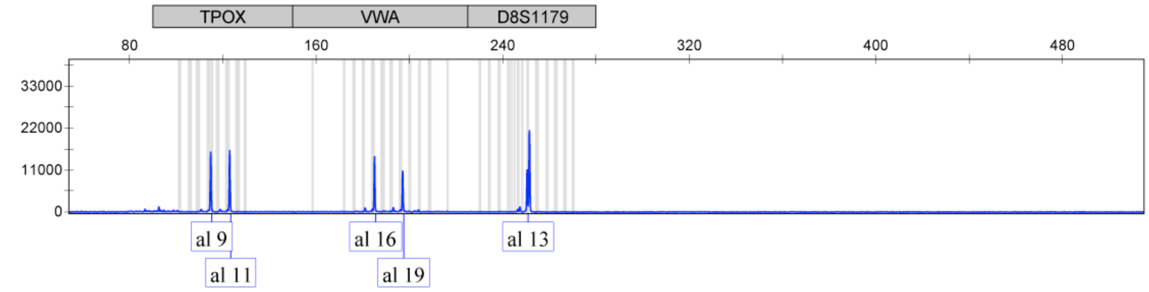

Applied Biosystems
GeneMapper 4.0

Cell Line Authenticaion-4

Sample File	Sample Name	Panel	OS	SQ
65_A09_CellLineAuthentication-5-1019.fsa	ARPE-19	STR Profile 4-dup		

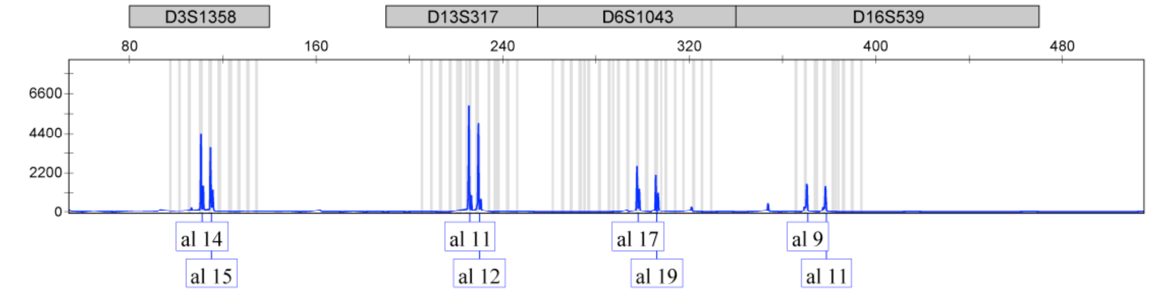

Sample File	Sample Name	Panel	OS	SQ
72_H09_CellLineAuthentication-5-1019.fsa	ARPE-19	STR Profile 5-dup		

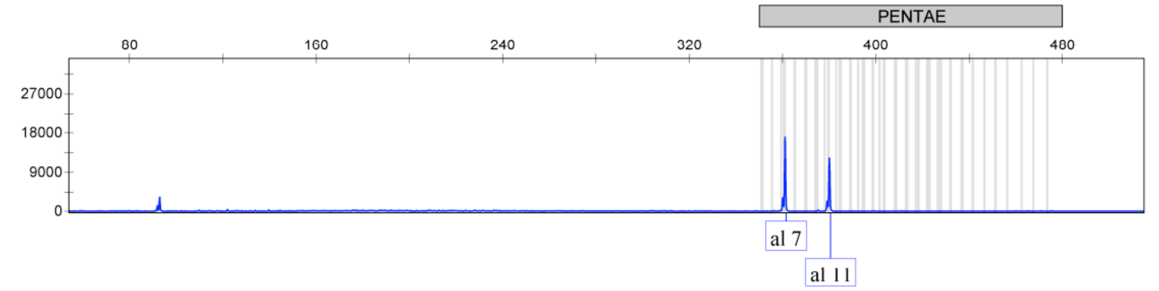

Sample File	Sample Name	Panel	OS	SQ
79_G10_CellLineAuthentication-5-1019.fsa	ARPE-19	STR Profile 6-dup		

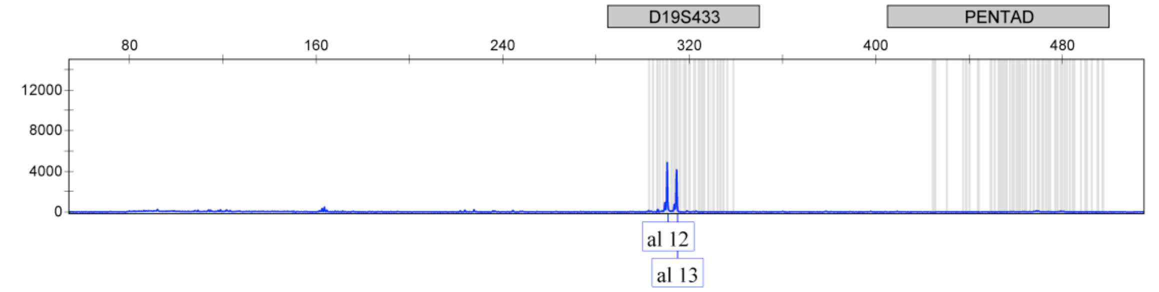

Applied Biosystems
GeneMapper 4.0

Cell Line Authenticaion-4

Sample File	Sample Name	Panel	OS	SQ
78_F10_CellLineAuthentication-1020.fsa	ARPE-19	STR Profile 6-dup		

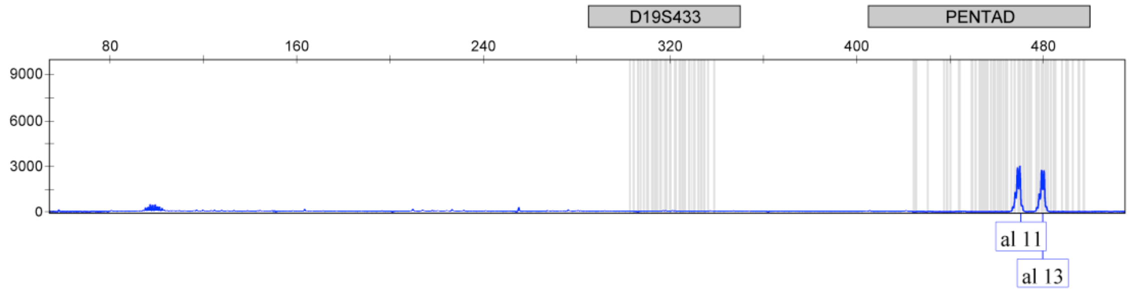

Supplement: Supplementary file 2 — Supplementary Material 2-ARPE [file 12020_2019_1921_MOESM2_ESM.pdf]
